# Robert Provine: the critical human importance of laughter, connections and contagion

**DOI:** 10.1098/rstb.2021.0178

**Published:** 2022-11-07

**Authors:** Sophie K. Scott, Ceci Qing Cai, Addison Billing

**Affiliations:** Institiute of Cognitive Neuroscience, University College London, London, London WC1N 3AR, UK

**Keywords:** laughter, behavioural contagion, vocalizations

## Abstract

Robert Provine made several critically important contributions to science, and in this paper, we will elaborate some of his research into laughter and behavioural contagion. To do this, we will employ Provine's observational methods and use a recorded example of naturalistic laughter to frame our discussion of Provine's work. The laughter is from a cricket commentary broadcast by the British Broadcasting Corporation in 1991, in which Jonathan Agnew and Brian Johnston attempted to summarize that day's play, at one point becoming overwhelmed by laughter. We will use this laughter to demonstrate some of Provine's key points about laughter and contagious behaviour, and we will finish with some observations about the importance and implications of the differences between humans and other mammals in their use of contagious laughter.

This article is part of the theme issue ‘Cracking the laugh code: laughter through the lens of biology, psychology and neuroscience’.

## 1. Laughter

Human laughter is a non-verbal emotional expression, characterized by sequences of regular short bursts of exhalations [1–3]. Laughter is recognized cross-culturally [4], and humans are not the only animals that laugh: vocalizations associated with tickling have been described in other apes [5] and in rats [6]. Play vocalizations, which may form evolutionary precursors to laughter, are found in a very wide range of animals, including kea parrots, cows and weasels [7]. In humans, laughter is one of the earliest vocalizations produced by infants [8]. Laughter is rich in information: adult listeners can distinguish between spontaneous and communicative laughter [9], and listeners can determine how close two talkers are by the sound of their laughter [10]. Listeners can also tell two talkers apart from their communicative laughter, but not their spontaneous laughter [11], suggesting that communicative laughter may be controlled (at least in part) via the volitional speech motor network [12], while spontaneous laughter may be controlled entirely by the evolutionarily older involuntary vocalization network. In reality, much naturally occurring laughter is likely to be a mix of both types [13].

Laughter is a fast-growing field of academic research, and we as a field owe a great debt of gratitude to Robert Provine, who made many fundamental contributions to the basic science of laughter, and who has inspired much of the more recent work. This paper will use some naturalistic laughter to explore his contributions in the context of more recent work, with a particular emphasis on the implications of his work on laughter and contagion.

## 2. Naturalistic studies of laughter

One of the most difficult aspects of the scientific study of laughter is that it is very hard to get people to produce laughter under laboratory conditions. Robert Provine had a lot of insight into this problem, and he made many of his most insightful observations about laughter from studying humans in natural interactions. Provine drew direct analogies with Jane Goodall's studies of chimpanzee behaviour [14], which moved primate research out of artificial, captive environments (e.g. [15]). Laughter, he argued, needs to be studied in ecologically valid environments, and certainly our experience of working with laughter in the laboratory has revealed that one cannot simply bring an adult participant into the laboratory and expect them to be able to laugh to command [16,17]. Unlike emotions like fear, which can be pretty easily generated in the laboratory by (for example) telling people to expect a small electric shock (e.g. [18]), people need certain social and emotional contexts to be appropriate for laughter to be possible or acceptable, and these contexts can be hard to recreate in the laboratory.

Our example is a quasi-naturalistic example of laughter. Thirty years ago, on 9 August 1991, two British Broadcasting Corporation (BBC) broadcasters, Brian Johnston and Jonathan Agnew, were reporting on a cricket test match between England and the West Indies. In the match they were summarizing, the English player Ian Botham had tried to jump over the wicket but just caught a bail (part of the wicket) with his leg, and thus was out of the game. Brian Johnston was describing this, when Jonathan Agnew interrupted with a joke:

**JONATHAN AGNEW**: And he knew exactly what was going to happen. He tried to step over the stumps and just flicked a bail with his right leg.
**BRIAN JOHNSTON**: He more or less tried to do the splits over it, and unfortunately, t-the inner part of his thigh must have just removed the bail.
**AGNEW**: He just didn't quite get his leg over *(pause)*
**JOHNSTON**: (*audibly smiling*) Anyhow, he – he *(pause)* did very well indeed, batting 131 min and hit three fours. And um then we had L-Lewis playing extremely well for h-his 47, not out. (*sound of Agnew laughing*) Aggers, do stop it. (*sound of Agnew laughing*) er and (pause) he was joined by DeFreitas (*sound of Agnew laughing*), who um was in for forty minutes, a useful little partnership there, they put on thirty five in forty minutes and then he was caught by Dujon off Walsh, *(pause)* um *(pause)* Lawrence *(pause)* huh always entertaining, batted for thirt – thirty five (*Johnston starts to laugh*) – thirt *(pause)* thirty five minutes, hit a four over the wicketkeeper's … h-(*loud wheezing laugh*)
(*BOTH LAUGH*)
**JOHNSTON**: Aggers, for goodness sake, stop it….
(*BOTH LAUGH*)
…hit a f-
(*BOTH LAUGH*)
**AGNEW**: yes Lawrence *(pause)* Lawrence *(pause)* played ….
(*BOTH LAUGH*)
*…*extremely well…
(*BOTH LAUGH*)
**JOHNSTON**: *(very high pitched voice)* He hit a ….
(*BOTH LAUGH*)
…he hit a four over the wicketkeeper's head, and he was out for nine…
*(BOTH LAUGH)*
**JOHNSTON:** *(talking through laughter, Agnew laughing in the background)*. And Tufnell came and batted for twelve minutes and was caught by Haynes off Patterson for two *(pitch of voice starts to normalize, laughter starts to fade)*, there were fifty-four extras and England were all out for four hundred and nineteen I've stopped laughing now.
(https://en.wikipedia.org/wiki/Jonathan_Agnew#%22Leg_over%22_incident, original recording can be found here Archive: Test Match Special - ‘Leg over’ incident ft Brian Johnston and Jonathan Agnew).

**Figure 1.**
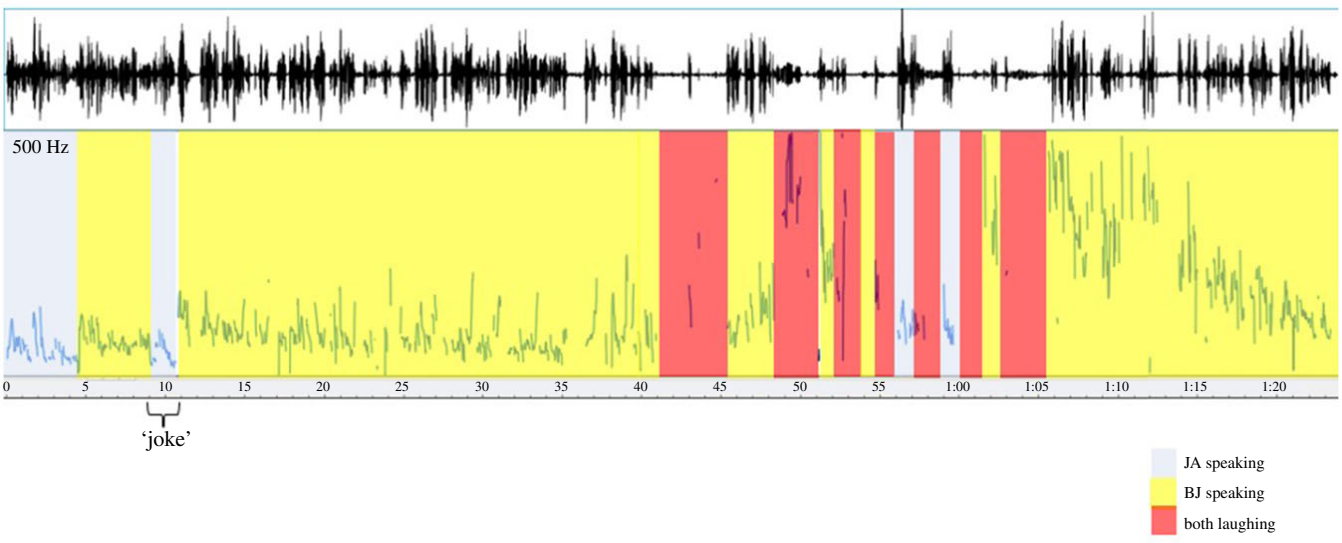
The top panel shows the oscillogram of the speech, and the lower panel shows the pitch profile, estimated in Praat. The different coloured panels delineate who was speaking or laughing. Note the brief period of silence after the ‘joke’.

Figure 1 shows the pitch profiles of the speech and laughter sequence of the sequence annotated to show when either man is speaking, and when they are both laughing.

The laughter is prompted by a clear joke ‘He just didn't quite get his leg over’: this is arguably not an especially funny joke. Agnew was making a reference that has or had a direct implication of sexual intercourse, but the comment had a clear intent to be humorous. This joke may have been made because the tone of the whole broadcast was jovial, with Brian Johnston having previously made several attempts to make the more junior Jonathan Agnew laugh (https://www.bbc.co.uk/programmes/p09v432k). This may be the reason why, when Agnew makes his joking comment, both men audibly react (there is a pause) then Agnew can be heard laughing ‘off mic’ (in the background), while Johnston continues talking. However, as soon as the joke is made and Agnew starts to laugh, Johnston's speech becomes noticeably more dysfluent, with several pauses and repetitions, and his voice is immediately raised in pitch (figure 1), which strongly suggests that Johnston has started to smile. Smiling alters the spectral shape of speech and also raises the pitch of the voice [19], which is why we can hear a smile from the voice alone. Smiles are (or can be) precursors to laughter [20,21], and here the smile may be initially an acknowledgement of the joke itself, or a behaviourally contagious response to Agnew's audible laughter (hence perhaps ‘*Aggers, do stop it*’): of course, it may be both. Johnston keeps talking, somewhat dysfluently. Around 30 s after the original joke, however, Johnston's articulation starts to break up on the phrase ‘thirty five minutes’, including a 5 s pause, in which he and Agnew can be heard laughing (figure 1). He then manages to say ‘he hit a four over the wicket-keeper's hh-‘ but when he reaches the glottal fricative /h/, he produces a long, wheezing laugh, followed by another plea to Agnew to stop laughing (*Aggers for goodness sake stop it*), followed by more laughter. Agnew joins in with the laughter. From the pitch profile in figure 1, the pitch of Johnston's voice rises a great deal once he has started to laugh. Notably, in none of these ostensible demands that Agnew stop laughing, does Agnew stop laughing, nor does Johnston sound at all upset or angry: his vocal pitch is still raised, and his tone arguably more the sound of someone complicit in the laughter, rather than someone who is demanding someone else actually cease laughing. Indeed, perhaps Johnston is also seeking to explain his own laughter to the listeners. At this point, Agnew tries to speak again, presumably to cover for Johnston (silence, or ‘dead air’ is to be avoided in live radio broadcasting), who manages to say ‘yes Lawrence….Lawrence played extremely well…’ with Johnston continuing to laugh audibly: Agnew's voice then fades into laughter while Johnston (finally) manages to say ‘he hit a four over the wicket-keeper's head and he was out for 9’. After this, his speech is still interleaved with laughter, but the pitch of his voice starts to come back down and he even says ‘I've stopped laughing now’, possibly as much for the audience as for the BBC producer who by this stage was now apparently standing in front of both men, glaring at them. The BBC does not like broadcasters to show emotion in the voice, and this full-on laughter may have been considered to be a little unseemly.

Following the initial live broadcast, this laughter episode proved very popular, featuring on the following day's BBC sports reports, and earning its own radio programme 30 years later (https://www.bbc.co.uk/programmes/p09v432k). There were many anecdotal stories of cars pulling to the sides of the road, the drivers unable to drive safely as the clip had made them laugh so much. On the 30-year anniversary programme, it was claimed that the cricket laughter excerpt is the second most popular spoken word sequence to have been selected on Desert Island Discs, the longest running radio programme in which guests choose eight tracks (normally musical pieces) to take to a fictional desert island.

This short radio clip is still highly popular in the UK, and it is typically described as ‘hilarious’ and ‘the funniest thing I have ever heard’. However, it is worth considering why this whole sequence is regarded so fondly and considered to be so amusing: again, the research of Robert Provine and others can help us understand this.

### 2.1. Social laughter—30 times more likely to laugh

The first factor that this clip demonstrates is that laughter is a highly social phenomenon—people who are on their own do not commonly start to laugh like this. Of course, this was one of Robert Provine's major contributions to the field of laughter research—demonstrating empirically that people are vastly more likely to laugh in the presence of other people than when on their own [22]. This study used a diary method in which people recorded episodes of laughing, smiling and talking, and all three were much more common in a social setting than when alone. Social contact increases all three social behaviours. In addition to the social context for laughter (and talking and smiling), there was a marked effect of time of day on laughter episodes. While all three behaviours fall away completely while the participants were asleep (along with almost all other volitional actions, like walking and playing the guitar), laughter was attenuated before sleep, and even more so immediately after sleep. This suggests that physiological/affective states also lead to modulations in the likelihood of laughter occurring, in addition to social context. Following sleep, it is possible that the reduction in alertness and cognitive abilities associated with sleep inertia markedly reduce the amount of laughter being produced (e.g. [23]). Sleep inertia is a transitory period between sleep and full alertness and is marked by a desire to return to sleep: at the same time as sleep inertia is having its effects on cognition and alertness, the cortisol awakening response is occurring, which can result in an unpleasant affective waking state. Either or both these mechanisms may be suppressing laughter when waking up. Other physiological changes can reduce the production of laughter: anxiety inhibits tickling responses and (presumably) laughter vocalizations in rats [24]. In humans, people with social anxiety report an increase in laughter (along with enjoyment and the feeling of acceptance) when they consumed a moderate amount of alcohol, an anxiolytic [25].

The role of social context in laughter has been extended to children—for example Addyman and colleagues found that 3- to 4-year-old children were eight times more likely to laugh at humorous material with other children around them than if they were on their own [26].

Provine and Fischer's finding of a dominant role for social and emotional context in priming laughter [22] was a critical step in delineating the ways that positive emotions interact with social and cultural factors. For example, recent work has shown that social context has a strong effect on the enjoyment of activities, demonstrating that it is more fun to do fun things with other people than on your own [27]. Notably this effect was reduced in people who reported higher levels of loneliness, consistent with a role for social interactions in the maintenance of laughter/social skills [28].

The relationship between social contact and positive emotions seems to be stronger than the link between social contact and negative emotions. For example, loneliness is associated with reduced smile mimicry, as measured by facial electromyography (EMG) of the zygomaticus major (smiling) muscle, while people look at International Affective Picture System photographs. However, in the same study, facial EMG of the corrugator supercilii (frowning) muscle showed no effect of loneliness—being equally engaged across all participants [29]. This link between positive emotions and social bonding supports Robin Dunbar's suggestion that human and primate social bonding is driven by endorphins [30]. Dunbar argues that the mechanisms which drive our social bonding are predicated on shared activities that are enjoyable—such as laughing, singing, dancing and eating, and which are associated with increased uptake of endogenous endorphins. This theory could predict that a lack of social engagement would have more selective effects on the processing of positive emotions than on negative emotions. Thus, although behavioural contagion of emotional states is extremely important in human interactions [31], it is the sharing of *positive* emotions that is critically important in social cohesion. Can we determine what the direction of causality is here? Do people who share positive emotions feel closer, or do we share more positive emotions with those we feel close to? There is some evidence that social contact interacts with emotional responses, which in turn enhance the social cohesion. As noted earlier, there is a negative relationship between loneliness and empathy, although this is moderated by individual differences in trait empathy [28].

The influence of social contact on positive emotional responses can also be seen at a cultural level, where there is evidence that it is the social environment that can drive differences in emotional responses. Countries that have a more varied history of migration are cultures where smiles and laughs are more easily and accurately decoded [32]. There is also a positive correlation between historical heterogeneity of populations and the *amount* of smiling and laughing that they report [32]. This can even be mapped within one country: US states show a positive correlation between rates of historic migration and the frequency of laughing and smiling. The argument here is that when there is greater heterogeneity (linguistically and culturally) in a group of people, a greater reliance on non-verbal expressions of emotion becomes important, and socially important emotional expressions, such as laughter, become more appropriately frequent. This key link between social context and laughter also feeds into Robin Dunbar's argument that laughter may form a very important role in the evolution of humans, by forming a positive bonding behaviour that is not reliant on language, and indeed, may predate it [33].

### 2.2. Incapacitated by laughter

The next point we can see from this laughter example is the extent to which the laughter overwhelms the two broadcasters. Provine was of the view that laughter can be a highly stereotyped, automatic behaviour [1], and this bears some similarity to the claims that we and others have made that some laughter can be highly spontaneous and involuntary: such laughter will occur even if the speaker is trying to inhibit it, and it will disrupt speech and breathing. Anatomically, it will probably be associated with the midline vocalization network that controls highly automatic and reactive emotional vocalizations [34,35]. However, this does not necessarily imply that communicative laughter, which Provine showed can be very precisely timed in conversations (spoken or signed) [36] is always under central attentional control. Indeed, much communicative (or ‘non-Duchenne’) laughter [13] may well be a mix of the two anatomical systems. Further work on the neural control of different kinds of laughter production will be able to answer this question in more detail, but it is worth noting that a lot of speech production can be highly automatic and over learned, but still be under a degree of voluntary control, from a neural perspective [37]. The same could be the case for laughter.

### 2.3. Social laughter and behavioural contagion

Two further highly important features of laughter that Provine identified are that laughter is rarely associated with jokes [38], and that laughter production is often associated with a strong degree of behavioural contagion [20]. Through observational studies, Provine noted that laughter is commonly associated with comments and statements that do not have any obvious humorous intent [38]. One of Provine's key discoveries is that human laughter is frequently used in a highly communicative way: the most common source of laughter in a conversation is usually the person who is talking at any one time (especially if they are female) [38]. Laughter is used to respond to overt humour, but it is also used to mark (or seek) affiliation, affection, agreement, understanding and recognition, in conversational settings [38]. We do laugh at things that we find funny, but most of the laughter we produce has nothing to do with humour. Similarly, Provine found that laughter is highly behaviourally contagious, with many laughter episodes occurring simply because someone else has laughed [20]. These contagious laughs are also highly social—people are much more likely to catch a laugh from someone they know than from a stranger [39].

There are several key points here that can help us understand the cricket laughter. The first is that the joke really is not very funny. It is beyond the remit of this article to delineate a comprehensive theory of humour, but it is certainly the case that the joke rests on a pretty simple ambiguity: Ian Botham tried to get his leg over the wicket, and ‘leg over’ is also a crude(ish) reference to male sexual behaviour that was popular (within reason) in the UK in the 1980s and 1990s. The humour may stem partly from the double meaning, where one meaning is a bit naughty (a benign violation) (e.g. [40]) and partly from the perceived intent, the desire to be funny. However, almost immediately the two men are, we suspect, not laughing because the joke is so funny, but because their laughter is itself highly behaviourally contagious [20]. They are each laughing, because they are both laughing. Agnew starts laughing first, and consistent with Robert Provine's arguments, the behavioural contagion of laughter is strong: within 30 s of starting to speak after the joke, Johnston completely stops being able to talk.

Provine made a strong case for the importance of behavioural contagion [41], extending beyond laughter, demonstrating that it was a very common cause of human actions: many of the yawns and laughs that humans produce are happening solely because of behavioural contagion. This can be extended to other behaviours like scratching, coughing and blinking [31]. What is the role of these mirroring behaviours that can be so powerful? There are some key points to be made about behavioural contagion: behavioural contagions are learned behaviours. Babies do not catch laughter contagiously. Second, as noted above, behavioural contagion is social (e.g. [39]). If Johnston and Agnew had been strangers, they may well not have laughed like this. Once they have been acquired, behaviourally contagious actions are often carried out in a largely unconscious way, such that people may often not realize that they are, for example, catching a laugh from someone else—they know that they are laughing but may not realize why. Behavioural contagion is also found in other animals, where it is also associated with social factors—thus contagious yawning is found in animals that have higher levels of social affiliation, such as chimpanzees and budgerigars [42]. Contagious scratching is common in other apes—for example in orangutans, self-scratching is a stress response, and contagious scratching seems to reflect an acknowledgement of this stress [43].

However, although a range of behaviourally contagious actions are found across different animals, there is a striking exception. Laughter is highly contagious in humans, and humans will even laugh if they hear recorded laughter (or show a priming response if they remain silent) [44]. However, despite the commonality of behavioural contagion in nature, and many other similarities between human and ape laughter, there are no clear reports of purely contagious laughter in any animals other than humans. Chimpanzees do laugh after hearing another chimpanzee laugh during play, in a manner that is distinct from their spontaneous laughter [45]. However, they appear to need to be physically engaged in play with another chimpanzee to produce laughter that is contingent on the laughter of that other chimpanzee. In a study looking at chimpanzee's responses to a humanoid robot that either moved randomly or imitated the chimpanzee [46], the chimpanzees were sensitive to the imitations and responded with greater attention, as well as attempts to engage the robot in interactions. However, when the robot ‘produced’ sounds, which were the sounds of laughs and screams from unfamiliar chimpanzees, the chimpanzees did not respond differently to either class of sounds. Notably, they did not laugh at all in response to any of the laughter sounds. The authors note that for laughter to be contagious for chimpanzees, ‘Perhaps a real and familiar social partner and the natural playful context must be present for chimpanzee laughter to induce positive responses in conspecifics, as found in natural social play of chimpanzees' [46, p. 594]; in contrast they suggest, perhaps the acoustic nature of human laughter makes it more effective, or human neural responses to laughter effect this contagion.

We would like to suggest a different possibility: a marked cognitive difference between humans and other apes which means that humans can provoke laughter and respond to this provocation *at a physical distance*. Thus, while physical tickling is a universal factor that makes infant humans, chimpanzees, gorillas, orangutans and rats laugh, it is also that case that right from the first emergence of laughter, non-physical contact acts can also make infant humans laugh. A study by Sroufe & Wunsch [8] showed that while physical acts (like tickling, kissing, jiggling overhead and bouncing on knee) were associated with babies' laughter at ages 4–6 months, so were ‘acoustic’ actions, such as lip popping, going ‘ahhhh’, social actions, like ‘I'm going to get you!’ and playing peek-a-boo, as well as silent visual actions, such as the mother shaking her hair. At older ages, 7–9 months and 10–12 months, babies start to laugh a great deal more in general, and while they still laugh at tactile and auditory actions, they start to laugh proportionally more at social and visual actions. The range of mother–baby interactions that lead to the babies' laughter are far more varied than just ‘tickling’ and in many cases involve no physical contact whatsoever. They all involve interactions with (in this experiment) their mothers, and playful intentions on the part of the mothers, but the majority of these are not seen as ways of eliciting laughter in infant apes. Perhaps human infants are able to represent playful intentions, directed towards themselves, as forming a social connection, which functions like playful physical contact. For example, playing and understanding ‘peekaboo’ relies heavily on the intentional hiding, and then revealing, of the parent's face and eye contact, and may rest on human's ability to infer intentions and connections from eye gaze (e.g. [47]). The important link into the contagious aspects of laughter is that laughter can be elicited from human infants, from a very young age, by physical contact but also from a variety of non-physical acts; and maybe this forms the basis of laughter contagion—babies learn to laugh contagiously, but maybe their ability to do so—for laughter to ‘jump the gap’ between humans—rests on this ability to feel a connection that does not require direct physical contact.

More studies are needed—it is hard to argue to an absence of evidence about contagious laughter in non-human apes—but it is also intriguing to consider another implication of the possibly unique nature of contagious laughter in humans—its role in humour. There is incomplete evidence about the production of, and recognition of, humour in non-human primates. Play is extremely common in mammals, and this includes primates, and play signifiers such as play face and laughter very frequently accompany primate play behaviours (e.g. [45]). However, there are very few demonstrations of humour in wild non-human primates—there are many examples of teasing (in wild and captive animals), which is usually aggressive, sometimes playful, but rarely accompanied by laughter (though it may not have been recorded) [48]. Teasing may be an important precursor to humour but in and of itself it does not function like human humour (including humorous teasing), which is much more commonly responded to with laughter. Taking a more precise model of different kinds of humour in apes [49], there are almost no examples of unambiguous humour in non-human apes, in the wild. There are more examples of humorous intent, such as playful misdirection, in captive apes. For example, the signing gorilla Koko was reported to playfully get words ‘wrong’, often accompanied by a play face (discussed in [49]). Notably, however, this was not reported to be accompanied by laughter on the part of Koko, nor are there reports of Koko responding to jokes made by the human experimenters (though again, here, we are arguing to an absence of evidence). It would also be important to unpack the actions of the humans in these scenarios, as they may well be contributing to the emergence of humorous intentions: captive chimpanzees have been reported to deliberately lure humans into paying attention to them, before throwing faeces at them [49]. Normally an act of aggression in the wild, this behaviour has been argued to be escalated in captive chimpanzees by the laughter of the humans, who laugh, even as they flee the faecal onslaught. The implication is that the humans' laughter response encourages this behaviour, although the chimpanzees themselves are not reported not laugh then they launch their attack.

This paper is not setting out a comprehensive theory of laughter, and what we find funny, but maybe Provine's insights into the behavioural contagiousness of laughter in humans sets the context for laughter to spread around human groups without the requirement of physical contact, which may make it spread faster and enable more people to share in the laughter than would be possible in physical play. Perhaps the ability of laughter to ‘jump the gap’ between humans also potentiates the basis for their finding playful non-contact acts to be funny, and to laugh. Also, the laughter may amplify the perceived humour: we have even shown that when people rate how funny jokes are, we rate the jokes as funnier if they are followed by laughter, and the more spontaneous the laughter, the funnier it makes the joke [50].

Circling back to the ‘leg over’ cricket commentary laughter between Jonathan Agnew and Brian Johnston in August 1991, we can find that many strands of laughter research that were initiated by Provine help us understand what is happening in that interaction. A social context is increasing the likelihood of laughter, the laughter is overwhelming and uncontrollable, and very quickly they are not laughing because the joke is especially funny, but because they are both laughing—in other words, the laughter is behaviourally contagious. However, can Provine's research also lead us towards an understanding of why this clip has been so popular in the UK? and also, why it is considered to be ‘funny’? The joke, as we have seen, is pretty thin, and even given the huge individual differences in what people find funny [40], no one argues that this clip is amusing because the joke itself is funny. Instead, we suspect that the highly authentic, spontaneous nature of the laughter is what listeners are responding to, and the way that this laughter is shared between the two men. Strangers—especially strange men [39] would not laugh like this together. Perhaps when we listen to spontaneous laughter like this, we are hearing and responding to a glimpse of their emotional connection which this interaction reveals. Also, why is this thought to be funny, and not as touching or intimate? Well, as Provine found, we do not have much insight into laughter: we associate our own laughter with jokes and comedy even though we mostly laugh for social reasons: perhaps we reverse engineer this assumption when we laugh at clips like this—it makes us laugh, so it must be funny.

## Data Availability

This article has no additional data.
