# Correction to: ‘Robert Provine: the critical human importance of laughter, connections and contagion’ (2022) by Scott *et al.*

**DOI:** 10.1098/rstb.2022.0409

**Published:** 2023-01-02

**Authors:** Sophie K. Scott, Ceci Qing Cai, Addison Billing

*Phil. Trans. R. Soc. B* **377**, 20210178. (Published 21 September 2022) (https://doi.org/10.1098/rstb.2021.0178)

One of the authors' names was spelt incorrectly as Addsion Billing instead of Addison Billing.

This has now been corrected on the publisher's website.

